# A 58-Year-Old Woman’s Odyssey With Bilateral Nodular Opacities Comes to a Diagnosis

**DOI:** 10.7759/cureus.102329

**Published:** 2026-01-26

**Authors:** Angeliki Miziou, Romanos Ntiloudis, Panagiotis Kousidis, Anastasia Nikolaidou, Konstantinos I Gourgoulianis, Garifallia Perlepe

**Affiliations:** 1 Faculty of Medicine, School of Health Sciences, Department of Pulmonology, University of Thessaly, Larissa, GRC; 2 Faculty of Medicine, School of Health Sciences, Department of Neurology, University of Thessaly, Larissa, GRC; 3 Pathology Department, Theageneio Cancer Hospital of Thessaloniki, Thessaloniki, GRC

**Keywords:** benign metastasizing leiomyoma, bml, case report, lung metastasis, nodules

## Abstract

Benign metastasizing leiomyoma (BML) is an uncommon entity in which histologically benign smooth muscle tumors appear at distant sites, most frequently the lungs, in women with a history of uterine leiomyoma treated surgically. Despite its benign pathology, BML often presents radiologically in a pattern that mimics metastatic malignancy, resulting in substantial diagnostic uncertainty. Awareness of this condition is essential for avoiding unnecessary or overly aggressive interventions.

We describe the case of a 58-year-old woman and active smoker with a significant 40-pack-year history who was referred following the incidental discovery of multiple bilateral pulmonary nodules and two enlarging masses during routine coronary CT angiography. Remarkably, retrospective review revealed a 23-year history of slowly progressive pulmonary nodules. The patient was asymptomatic except for a chronic intermittent productive cough. Laboratory testing, including immunologic profiling and tumor markers, was unremarkable, apart from elevated CA-125 levels. Bronchoscopy and transbronchial needle aspiration demonstrated nonspecific fibrosis without evidence of malignancy or infection. Fluorodeoxyglucose-positron emission tomography (FDG-PET) imaging showed multiple photopenic nodules and two non-FDG-avid masses, further complicating the differential diagnosis, which initially included metastatic cancer, granulomatous disease, and primary pulmonary neoplasms. Given the increasing size of the dominant left upper lobe lesion and persistent diagnostic ambiguity, the patient underwent video-assisted thoracoscopic surgery with left upper lobectomy. Histopathological examination revealed intersecting bundles of bland smooth muscle cells without atypia or necrosis, while immunohistochemistry demonstrated strong positivity for SMA, desmin, caldesmon, and estrogen receptors, consistent with pulmonary benign metastasizing leiomyoma. These findings were correlated with the patient’s remote history of hysterectomy for uterine leiomyoma.

This case highlights the diagnostic challenges of BML, particularly when radiologic findings resemble metastatic malignancy over many years. Recognition of its characteristic clinical, imaging, and histologic features is essential to avoid misdiagnosis and overtreatment. Management should be individualized and may include surgical resection, hormonal therapy, or careful surveillance. Long-term follow-up is recommended due to the condition’s variable clinical course and potential for progression.

## Introduction

Benign metastasizing leiomyoma (BML) is a rare clinical entity characterized by the presence of histologically benign smooth muscle tumors at distant extrauterine sites, most commonly within the lungs. First described by Steiner in 1939, BML has since remained an infrequent but intriguing condition, with just over 160 documented cases in the literature [1]. The disease predominantly affects women of reproductive or perimenopausal age and is strongly associated with a prior history of uterine leiomyoma treated surgically, most often by hysterectomy or myomectomy. The temporal relationship between gynecologic surgery and the development of pulmonary nodules varies widely, ranging from several months to more than two decades, adding to the diagnostic complexity of this condition [2].

The true pathogenesis of BML remains uncertain, and multiple theories have been proposed. The leading hypothesis supports hematogenous dissemination of monoclonal smooth muscle tumor cells originating from the uterus, facilitated during surgical manipulation. Cytogenetic studies demonstrating chromosomal abnormalities such as trisomy 12, rearrangements of 6p, and balanced translocations lend further support to this mechanism. Hormonal influences are also thought to play a pivotal role, as most BML lesions express estrogen and progesterone receptors and often remain indolent until climacteric hormonal changes occur. Alternative hypotheses, including lymphatic spread, multifocal smooth muscle proliferation, and misdiagnosed low-grade leiomyosarcoma, have also been suggested but lack consistent evidence [3].

Clinically, BML is frequently asymptomatic and is commonly detected incidentally during imaging studies performed for unrelated reasons. When symptoms do occur, they are typically nonspecific, such as chronic cough, dyspnea, or chest discomfort. Radiographically, BML characteristically presents as multiple, well-circumscribed pulmonary nodules of varying size, often resembling metastatic malignancy. The absence of fluorodeoxyglucose (FDG) uptake on PET imaging may provide an important diagnostic clue, yet this finding is not exclusive and cannot rule out malignancy. Because of this overlap, the differential diagnosis of multiple pulmonary nodules in women with a history of uterine fibroids is broad and includes metastatic cancer, infectious granulomatous diseases, inflammatory disorders, and primary pulmonary neoplasms [4].

Given these diagnostic challenges, histopathological confirmation with immunohistochemistry remains the cornerstone of establishing a definitive diagnosis. BML lesions demonstrate the classic appearance of benign smooth muscle proliferation without mitotic activity, atypia, or necrosis, along with strong positivity for smooth muscle markers such as SMA, desmin, and h-caldesmon. Recognizing this characteristic profile is essential to prevent misdiagnosis and to guide appropriate management strategies, which may range from observation to surgical resection or hormonal therapy depending on disease extent and symptomatology [5].

In this report, we describe a case of long-standing, slowly progressive bilateral pulmonary nodules in a 58-year-old woman, ultimately found to represent benign metastasizing leiomyoma more than two decades after hysterectomy. This case highlights the diagnostic uncertainty, radiologic mimicry, and indolent natural history of BML, underscoring the need for awareness of this rare entity among clinicians evaluating pulmonary nodules in women with prior uterine fibroids.

## Case presentation

A 58-year-old woman, active smoker with a 40-pack-year history, was referred by her cardiologist following the incidental discovery of multiple pulmonary nodules and masses on coronary computed tomography (CT) angiography obtained during her routine annual screening. She denied dyspnea, chest pain, fever, night sweats, or weight loss over the preceding year, although she reported a persistent productive cough. The cough was intermittent without reporting triggering factors. Her comorbidities include hypertension, dyslipidemia, and hypothyroidism, managed with amlodipine, atorvastatin, and levothyroxine, respectively. Two decades earlier, she underwent a total hysterectomy with bilateral salpingo-oophorectomy due to uterine fibroids. Her occupational history revealed no exposure to industrial or environmental hazards, and she denied alcohol use, illicit drug consumption, and recent international travel.

At presentation, the patient was afebrile and hemodynamically stable, with a blood pressure of 110/60 mmHg, heart rate of 75 beats per minute, respiratory rate of 13 breaths per minute, and oxygen saturation of 98% on ambient air. Her breathing was unlabored, with no use of accessory muscles, and clubbing of the nails was absent. Chest wall palpation revealed symmetric expansion, preserved tactile fremitus, and normal resonance on percussion. Auscultation of the lungs detected no adventitious sounds. Cardiovascular assessment disclosed a nondisplaced apex beat, absence of thrills or murmurs, and no jugular venous distention or peripheral edema. Examination of the ear, nose, throat, and peripheral lymph nodes revealed no abnormalities or signs of edema or hemorrhage. The remainder of the physical examination was unremarkable.

**Figure 1:**
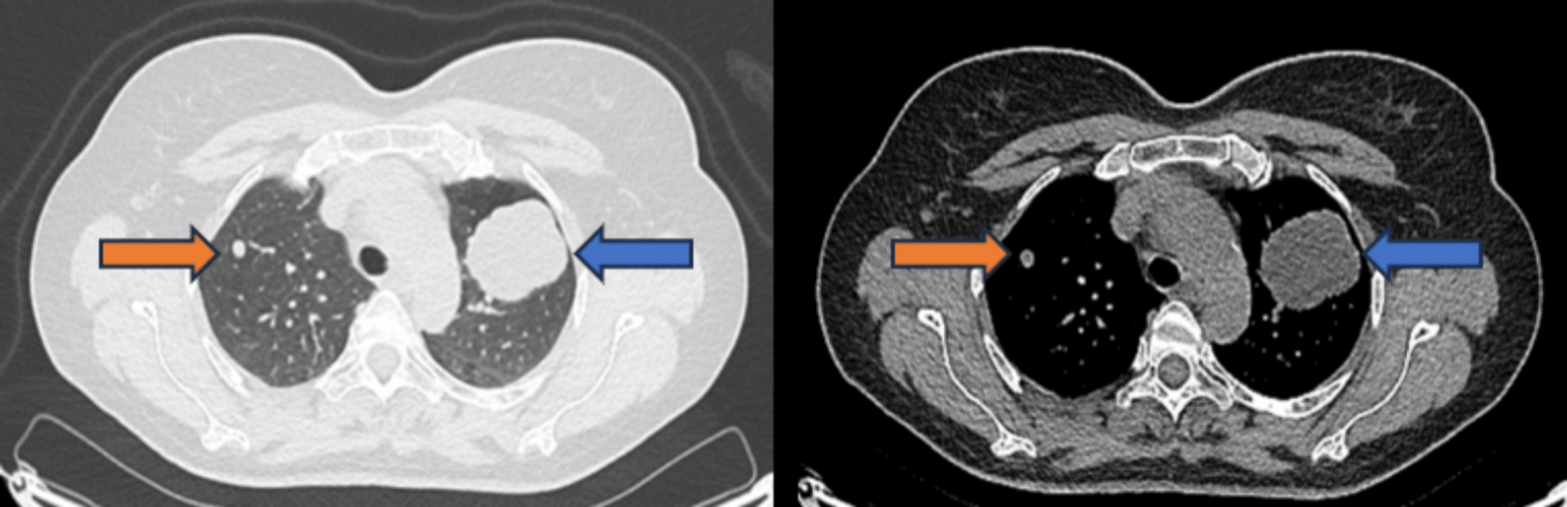
CT scan obtained at the time of the patient’s presentation showing a mass in the left upper lobe measuring 4.0 x 5.0 x 5.2 cm (blue arrow) and a nodule 0.3 cm in the right upper lobe (orange arrow)

The patient’s laboratory investigations were within normal ranges. Pulmonary function tests were also performed without impairment. A complete serum immunology panel, including serum amyloid, was taken with results within expected reference ranges, along with negative tumor markers, with the exception of a high level of CA-125. Based on the initial CT angiography, multiple peripheral nodules were detected, and two masses, with the bigger one in the left upper lobe measuring 4.0 x 5.0 x 5.2 cm (Figure 1). Flexible bronchoscopy did not show pathological endobronchial findings. Cytology tests and histopathology exam from the tissue obtained by transbronchial needle aspiration (TBNA) revealed non-specific focal fibrosis with no clues of malignancy. Acid-fast stain and cultures for Mycobacterium species and fungi were negative.

**Figure 2:**
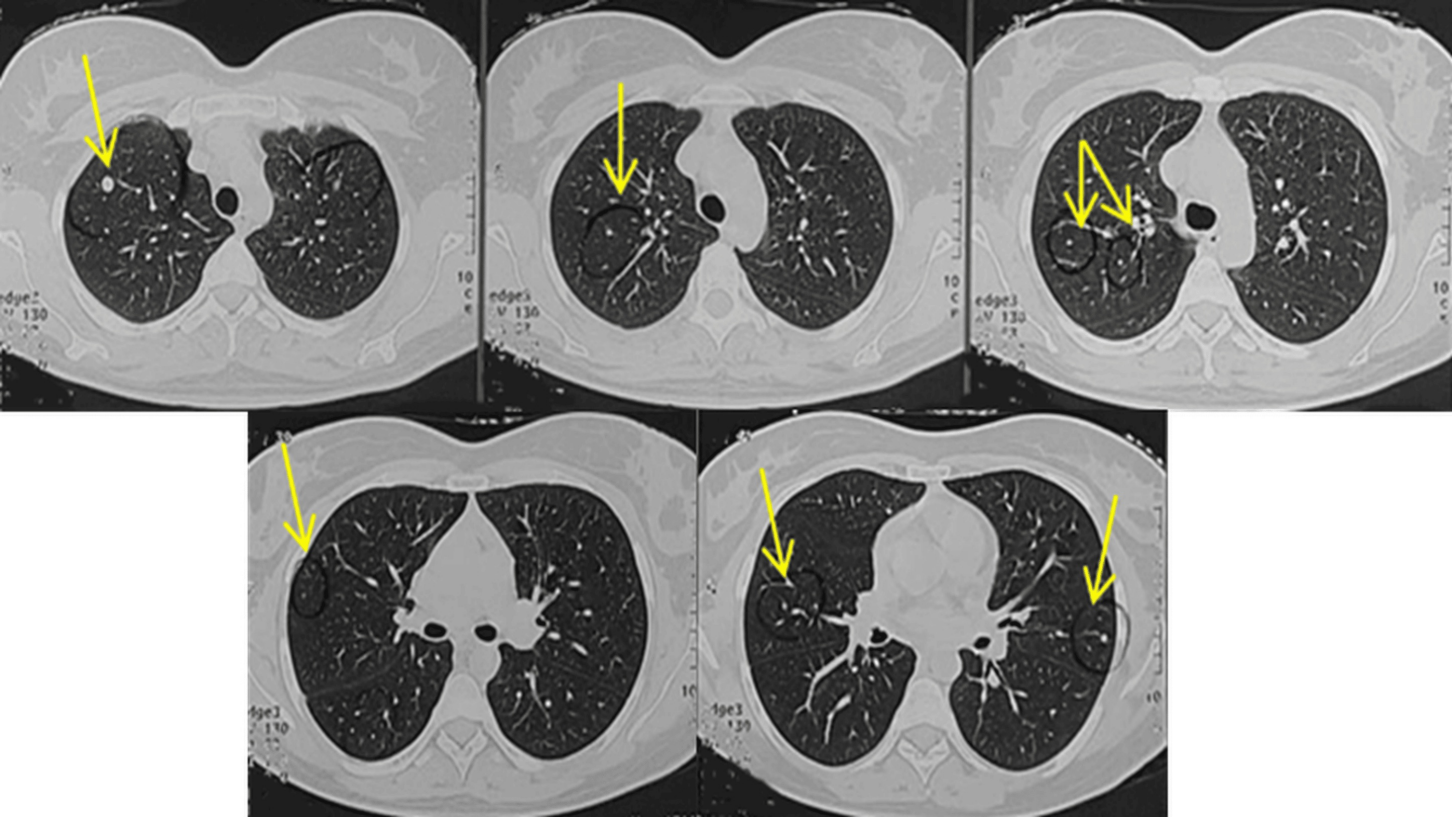
Axial chest CT images of the lungs obtained 23 years prior, showing multiple well-demarcated, round pulmonary nodules of varying sizes (yellow arrows)

**Figure 3:**
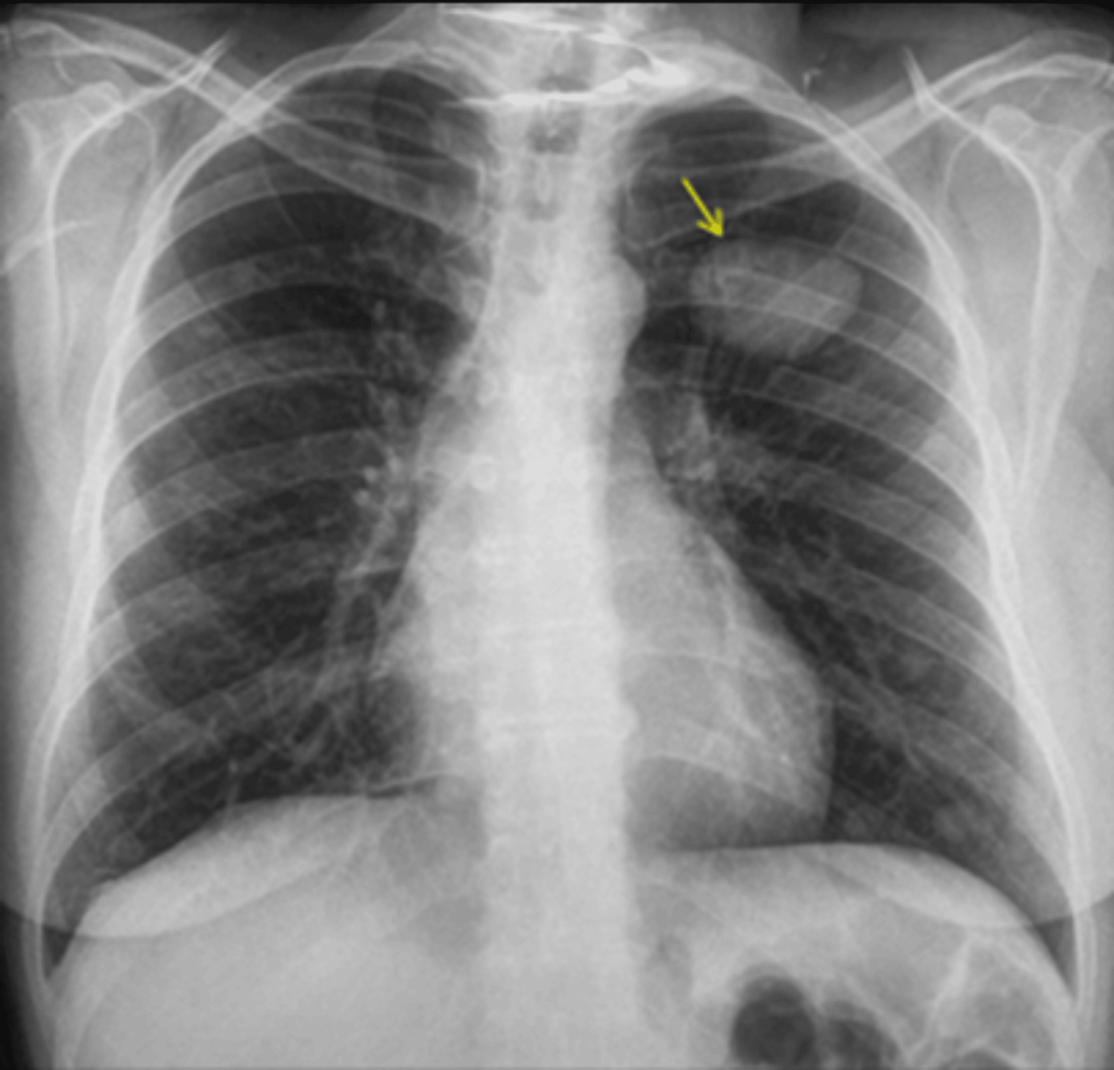
Frontal chest radiograph demonstrates bilateral lung nodules and masses. The largest lesion was noted in the left upper lobe, six years ago.

**Figure 4:**
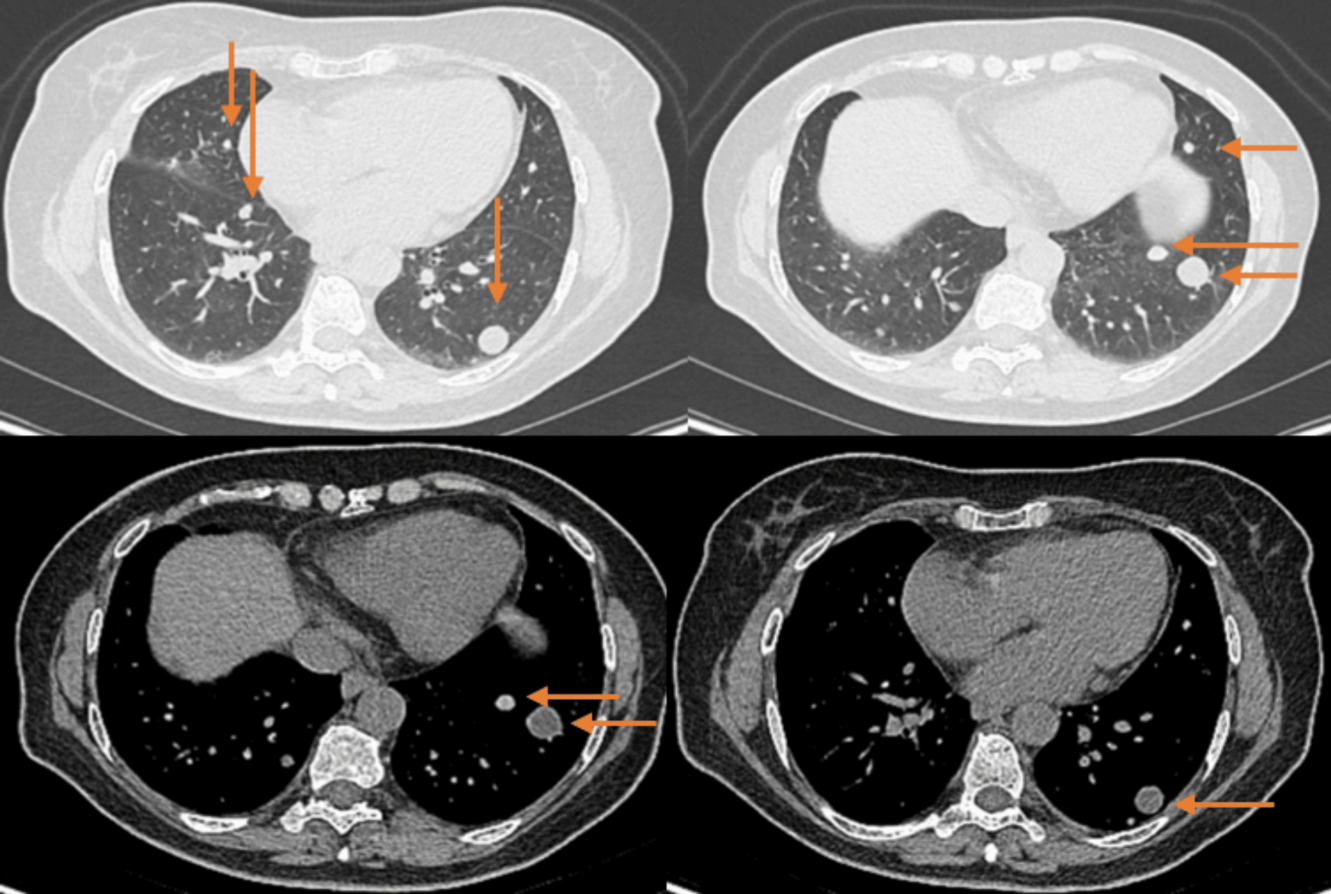
CT images show multiple, well-demarcated, round lung nodules of various sizes (orange arrows), two years ago

Previous radiological assessments have identified well-defined nodular lesions with smooth margins in both lungs, exhibiting progressive enlargement over the past 23 years (Figures 2-4).

**Figure 5:**
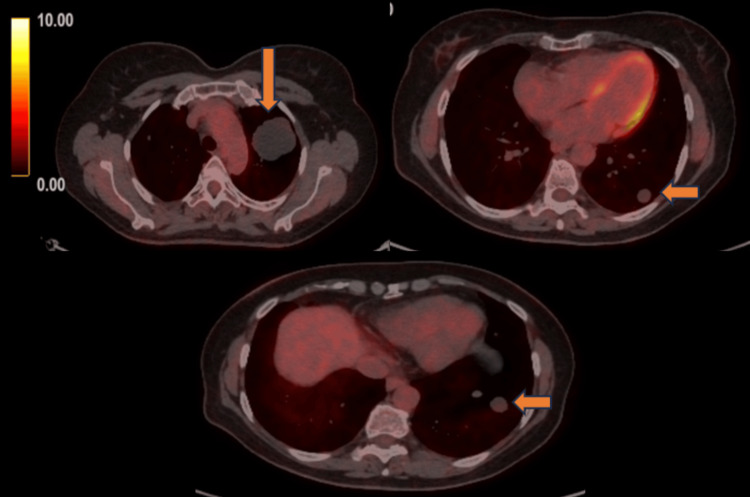
PET scan images obtained at the time of presentation show no visible FDG uptake in the multiple small lung nodules and masses (SUVmax 0.4–0.6) PET: positron emission tomography; FDG: fluorodeoxyglucose; SUVmax: maximum standardized uptake value

**Figure 6:**
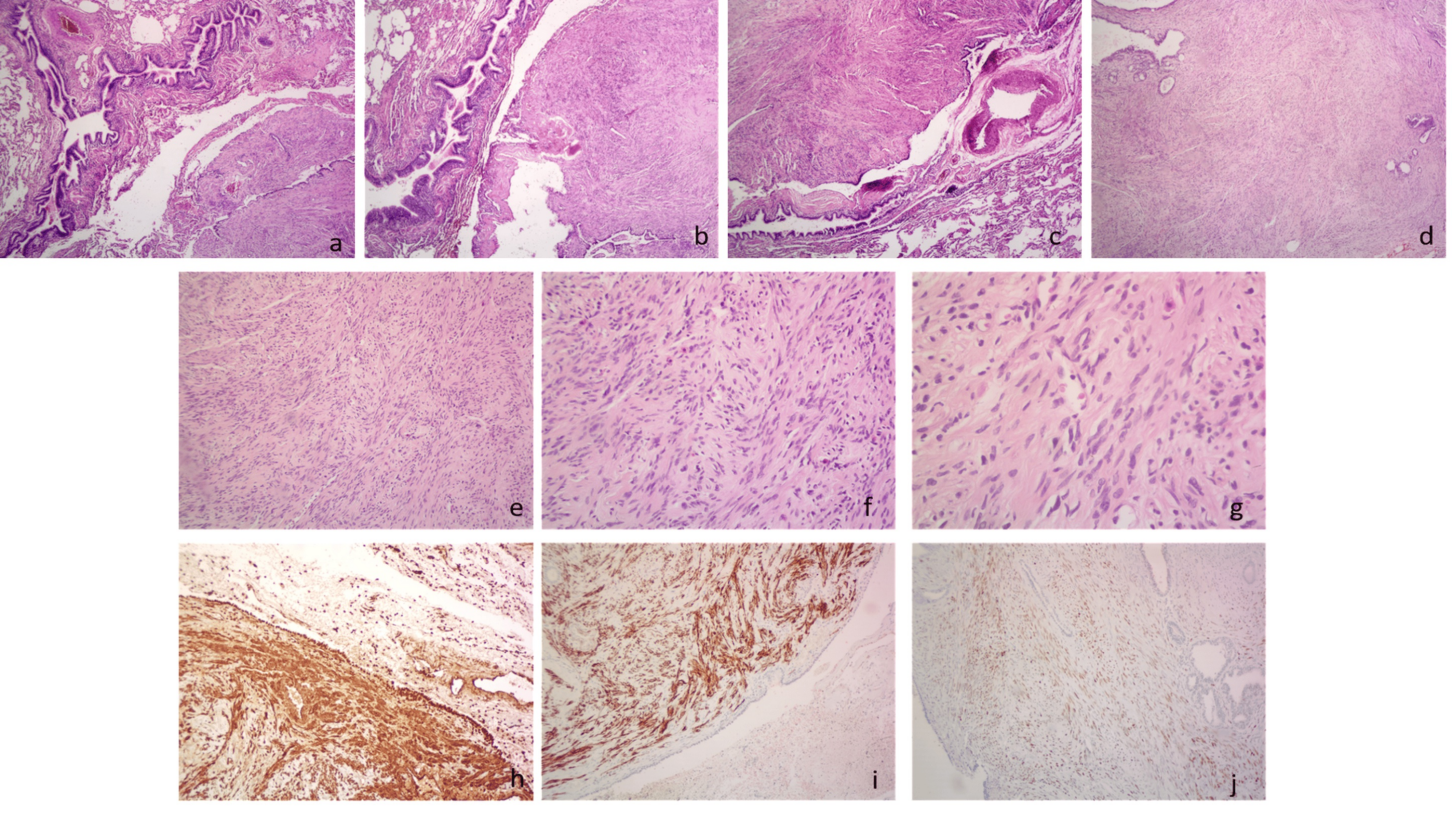
Histopathological and immunohistochemical findings of pulmonary benign metastasizing leiomyoma at the time of the patient’s initial presentation (a–c) Low-power histological sections (H&E, ×4) showing the tumor adjacent to the pulmonary parenchyma and bronchus (a, b), and the tumor adjacent to the pulmonary parenchyma, bronchus, and vein (c). (d) Low-power view (H&E, ×4) demonstrating the tumor with entrapped alveoli at the periphery. (e–g) Higher-power views showing features of pulmonary benign metastasizing leiomyoma with intersecting bundles of bland spindle cells (H&E, ×10 (e), ×20 (f), ×40 (g)). (h) Immunohistochemical staining for smooth muscle actin (SMA), ×10, showing diffuse positivity. (i) Immunohistochemical staining for desmin, ×10, showing diffuse positivity. (j) Immunohistochemical staining for estrogen receptor (ER), ×10.

A subsequent FDG-PET scan was performed to identify primary malignancy and possible metastatic lesions. Multiple photopenic small peripheral nodules (≤ 10 × 8 mm in diameter) and 2 lung masses (the largest one 52 × 54 mm in the left upper lobe) with no visible FDG uptake were noticed (Figure 5). At this stage, due to the size of the lesion in the left upper lobe and the pressure it exerted on neighboring structures, a biopsy was recommended by a thoracic surgeon. The patient underwent a left upper lobectomy, and multiple biopsies were taken by video-assisted thoracoscopy (VATS). Histopathological examinations showed a mesenchymal tissue neoplasm consisting of intertwining bundles of smooth muscle fibers without cellular atypia or necrosis. According to immunohistochemistry, the cells were strongly positive for smooth muscle actin (SMA), desmin, caldesmon, and estrogen receptors, focally positive for progesterone receptors, and negative for S100 protein, low molecular weight keratin (Ker 8/18), and CD34 (Figure 6).

The diagnosis of benign metastasizing leiomyoma correlated with the previous patient’s history of hysterectomy. Despite the benign nature of BML, the complexity of the condition warranted referral to an oncologist for further management, who provided expert guidance on long-term surveillance as well as possible future treatment options in case of progression.

## Discussion

Benign metastasizing leiomyoma (BML) is a rare, nonmalignant condition characterized by the spread of smooth muscle tumors, typically originating from the uterus, to distant organs, resulting in pulmonary, cardiac, nervous, skeletal, or soft tissue involvement, with the lungs being the most common extrauterine location (80% of cases). Since its first description by Steiner in 1939, over 161 cases with BML have been reported, the majority occurring in patients with prior hysterectomy or myomectomy for uterine leiomyoma [1-3].

The condition predominantly affects women in their late reproductive to early perimenopausal years, with a median age at diagnosis of approximately 46 to 47 years. The latency between uterine surgery and the detection of pulmonary lesions typically ranges from a few months to over two decades [4].

The pathophysiology of BML remains unclear, but it is believed that leiomyomas, often originating in the uterus, can gain access to the bloodstream or lymphatic system during surgery, allowing them to spread to distant sites. The most commonly accepted theory is the hematogenous spread of a monoclonal element in a benign smooth muscle tumor [5]. Chromosomal abnormalities can be found in 25% of such tumors, including balanced translocation, trisomy 12, or rearrangement of 6p. Other possible mechanisms include lymphovascular embolization, mesothelial-mesenchymal metaplasia, and metastases from misdiagnosed low-grade uterine leiomyosarcomas. Although the tumors remain histologically benign and do not show the invasive behavior of malignancies, they can still form metastatic-like lesions. The mechanisms behind this dissemination are poorly understood but may involve hormonal factors, particularly estrogen, which is thought to play a role in both the growth of leiomyomas and their potential to spread [5,6].

The clinical course of BML is usually indolent and asymptomatic, and when symptoms occur, presentations may include cough, dyspnea, or chest discomfort. Imaging studies, such as CT scans or chest X-rays, commonly reveal multiple, well-defined solid pulmonary nodules ranging from millimeters to several centimeters. Bilateral involvement is frequent, although solitary nodules or masses are possible. Cavitation or calcification is uncommon, as is mediastinal lymphadenopathy. Accordingly, PET-CT findings are characterized by low to absent FDG uptake, reflecting the benign and low metabolic nature of these lesions [7].

Differential diagnosis includes mainly metastases from malignant tumors and, more precisely, uterine leiomyosarcoma, other sarcomas, breast carcinomas, melanomas, or lymphomas. Furthermore, mycobacterial or fungal infections, sarcoidosis, rheumatoid nodules, and pulmonary amyloidosis can all mimic BML on imaging. Primary pulmonary leiomyoma and lymphangioleiomyomatosis (LAM) are also morphologically similar to BML [3].

Histopathological analysis is essential for diagnosis and illustrates well-circumscribed, spindle-cell proliferations characterized by bland, well-differentiated smooth muscle cells arranged in intersecting fascicles, with no evidence of mitotic activity, necrosis, or atypia. Immunohistochemical staining demonstrated strong positivity for smooth muscle markers, including SMA, desmin, and h-caldesmon, as well as estrogen and progesterone receptors, while showing a low Ki-67 proliferation index [6].

Given the benign nature of BML, treatment is tailored to its extent, symptoms, and hormone receptor status. For isolated pulmonary nodules, surgical excision remains the primary treatment choice when feasible. In cases with multifocal or unresectable disease, antiestrogen strategies with GnRH agonists and aromatase inhibitors have demonstrated the ability to induce lesion stabilization or regression. Additionally, progestins and selective estrogen receptor modulators (e.g., tamoxifen) have yielded promising results in individual cases. Observation with regular imaging surveillance is also acceptable for asymptomatic patients with indolent disease. No standardized management guidelines currently exist, and long-term follow-up is essential given the favorable but variable clinical course. The prognosis is generally favorable and does not typically result in significant morbidity or mortality [8].

## Conclusions

BML is a rare condition in which benign uterine smooth muscle tumors spread to distant sites, most often the lungs, which typically affects women of reproductive or premenopausal age, often appearing years after uterine surgery. Despite its benign nature, BML can present significant diagnostic challenges due to its clinical and radiologic resemblance to metastatic malignancy. Pulmonary lesions in BML are usually multiple, well-defined nodules found incidentally on imaging, and the disease generally follows a slow, indolent course with a favorable prognosis.

Treatment options for BML include resection of lesions, hormonal manipulation, or observation alone in a case-by-case scenario.
